# Beef, Chicken, and Soy Proteins in Diets Induce Different Gut Microbiota and Metabolites in Rats

**DOI:** 10.3389/fmicb.2017.01395

**Published:** 2017-07-27

**Authors:** Yingying Zhu, Xuebin Shi, Xisha Lin, Keping Ye, Xinglian Xu, Chunbao Li, Guanghong Zhou

**Affiliations:** Key Laboratory of Meat Processing and Quality Control, MOE, Key Laboratory of Meat Processing, MOA, Jiangsu Collaborative Innovation Center of Meat Production, Processing and Quality Control, Nanjing Agricultural University Nanjing, China

**Keywords:** NMR, gut microbiota, red meat, white meat, metabolites

## Abstract

Previous studies have paid much attention to the associations between high intake of meat and host health. Our previous study showed that the intake of meat proteins can maintain a more balanced composition of gut bacteria as compared to soy protein diet. However, the associations between dietary protein source, gut bacteria, and host health were still unclear. In this study, we collected colonic contents from the growing rats fed with casein, beef, chicken or soy proteins for 90 days, and analyzed the compositions of gut microbiota and metabolites. Compared to the casein group (control), the chicken protein group showed the highest relative abundance of *Lactobacillus* and the highest levels of organic acids, including lactate, which can in turn promote the growth of *Lactobacillus*. The soy protein group had the highest relative abundance of *Ruminococcus* but the lowest relative abundance of *Lactobacillus*. Long-term intake of soy protein led to the up-regulation of transcription factor CD14 receptor and lipopolysaccharide-binding protein (LBP) in liver, an indicator for elevated bacterial endotoxins. In addition, the intake of soy protein also increased the levels of glutathione S-transferases in liver, which implicates elevated defense and stress responses. These results confirmed that meat protein intake may maintain a more balanced composition of gut bacteria and reduce the antigen load and inflammatory response from gut bacteria to the host.

## Introduction

In recent years, excessive intake of meat and meat products has been suggested to be associated with some metabolic disorders (Tilman and Clark, 2014). Specifically, N-nitroso-compounds and heterocyclic amines, which were formed during cooking of red meat at high temperatures, could be critical factors for an elevated risk of mortality of colorectal cancer (Pan et al., 2012; Bastide et al., 2015). However, it is the fact that meat has many biological functions in terms of highly bioavailable nutrients, including essential amino acids, heme iron, and vitamins (Pereira and Vicente, 2013).

Food is a major factor that can shape gut microbiota (Subramanian et al., 2014). The gastrointestinal tract and residing bacteria have been shown to play a crucial role in extracting and metabolizing dietary ingredients (Muegge et al., 2011; Tyakht et al., 2012; Tang et al., 2013). There is about 12–18 g of protein entering into human colon every day, consisting of residual dietary proteins and endogenous enzymes secreted in the small intestine (Scott et al., 2013). Approximately 10% of ingested proteins can reach the colon, which is dependent on the type and amount of protein consumed (Cummings, 1997). The residual dietary proteins and endogenous enzymes are the main source of nitrogen for the growth of gut microbiota (Cummings and MacFarlane, 1991). Amino acids would become energy source in the distal colon (Hamer et al., 2012). Recent studies indicated that the metabolites derived from gut microbiota may have a certain impact on host health, for example, short chain fatty acids, especially butyrate, can be served as energy for host tissues (Flint et al., 2015). On the other hand, lipopolysaccharide (LPS), an endotoxin, can enter into the circulation and be bound to lipopolysaccharide-binding protein (LBP) in liver (Weiss, 2003; Zhao, 2013). The LPS-LBP complex further binds to CD14 receptor, which mediates the activation of macrophages to produce inflammatory cytokines (Lukkari et al., 1999).

Dietary intake influences the structure and activity of the trillions of microorganisms residing in the human gut (Wu et al., 2011; Muegge et al., 2011; David et al., 2014). For example, enterotypes were strongly associated with long-term diets, particularly protein and animal fat (*Bacteroides*) vs. carbohydrates (*Prevotella*; Wu et al., 2011). Gut microbiota and their metabolites showed differences after intake of casein and plant proteins (Geypens et al., 1997; Day, 2013; Rist et al., 2014), however, few data are available on how meat proteins affect gut microbiota and their metabolic activities. Rats and humans had similar gut bacterial communities, and thus rats are used as models to study the linkage between dietary proteins and gut microbiota (Ley et al., 2006). Our recent study showed that the rats fed with meat proteins have significantly different structure of gut microbiota in caecum from those fed with soy protein (Zhu et al., 2015). Nevertheless, it remains unknown how gut microbiota and metabolites in colon respond to different dietary proteins. In this study, we fed growing rats with casein, soy, beef and chicken proteins for 90 days and characterized colonic metabolites by using (Tilman and Clark, 2014) H NMR spectroscopy and gut microbiota by sequencing the V4-V5 region of 16S ribosomal RNA. Meanwhile, the levels of LBP mRNA/protein and CD14 mRNA/protein were measured to evaluate the bacterial endotoxin load to host. The association between colonic bacteria and metabolites, and its significance for host health were discussed.

## Materials and methods

### Animals and samples

Four-week-old male Sprague-Dawley rats (117 ± 10 g) were purchased from Zhejiang Experimental Animal Center (Zhejiang, China, SCXK9 <Zhejiang> 2008-00) and housed in a specific pathogen-free animal center (SYXK <Jiangsu> 2011-0037). After 7-day acclimatization (protein source: casein), the rats were assigned randomly to four formulated diets with casein, and proteins from beef, chicken and soy (*n* = 8 each group). Casein is the sole protein in standard rat diets recommended by the American Institute of Nutrition, and thus we set the casein group as the control. The formulated diets were prepared as described previously (Zhu et al., 2015). The animals were maintained individually in plastic ventilated cages and given water and diets *ad libitum* in a temperature and humidity (20.0 ± 0.5°C, 60 ± 10%) controlled room with a 12 h light/dark cycle. Experimental protocol involving animals was reviewed and approved by the Ethical Committee of Experimental Animal Center of Nanjing Agricultural University. All experiments were performed in accordance with the relevant guidelines and regulations of the Ethical Committee of Experimental Animal Center of Nanjing Agricultural University.

After 90-day feeding, all rats were sacrificed after 4 h fasting. The distal colonic contents were collected and transferred to two eppendorf tubes, then immediately frozen in liquid nitrogen and stored at −80°C for metabolomic and microbiota analyses.

### Microbiota and metabolomic analysis

Microbiota analysis was referred to our previous study (Zhu et al., 2015). Briefly, the caecal contents were collected, frozen in liquid nitrogen, and stored at −80°C before being analyzed. DNA was extracted from each sample using the QIAamp DNA Stool Mini Kit (NO. 51504, Qiagen, Germany) according to the manufacturer's protocol. The 16S ribosomal RNA (rRNA) gene from caecal contents was amplified with universal primers: F515 (5′-GTGCCAGCMGCCGCGG-3′) and R907 (5′-CCGTCAATTCMTTTRAGTTT-3′). The V4-V5 hypervariable region that is universal for nearly all bacterial taxa was applied for amplification. Purified amplicons were sequenced under the MiSeq platform (Illumina, San Diego, California, USA) according to the standard protocols in a commercial company (Shanghai Majorbio Bio-Pharm Technology Co., Ltd, Shanghai, China).

Metabolomic analysis was performed as follows: (1) 1.5 mL of ice-cold water was mixed with 300 mg of colonic samples, vortexed vigorously for 1 min, and sonicated at 4°C for 10 min. Then the samples were subjected to vortex shaking at 13,000 rpm for 30 min at 4°C and 450 μL of the supernatant was carefully transferred to a fresh eppendorf tube, and 50 μL of a standard buffer solution (ACDSS, Anachro Certified DSS Standard Solution, 4.136 mM) was added and vortexed vigorously for 10 s. The mixture was subjected to vortex shaking at 13,000 rpm for 20 min and a 480 μL of the supernatant was transferred to a NMR tube for subsequent NMR analysis. ^1^H-NMR spectra were obtained at 298 K under a Bruker AV III 600 MHz spectrometer (operating at ^1^H frequency of 600.13 MHz, Bruker Biospin, Germany) equipped with an inverse cyrogenic probe. A total of 32 scans were collected into 32 k data points for each spectrum with a spectral width of 8,000 Hz. Water presaturation for 1 s along with the recycle delay was applied for solvent signal suppression. All ^1^H-NMR spectra were processed and analyzed using the Chenomx NMR Suite Professional software package (version 7.7, Chenomx Inc., Edmonton, Canada). Qualitative and quantitative analyses of ^1^H-NMR spectra were performed by manually fitting spectral signatures from an internal database of Chenomx to each spectrum. The ACDSS was used as internal standard for chemical shift referencing (0 ppm) and quantification.

### Reverse transcriptase-polymerase chain reaction (RT-PCR)-based mRNA assay

A semi-quantitative RT-PCR assay was used to estimate the mRNA levels of LBP and CD14 in liver samples. Total RNA was isolated from liver samples using TaKaRa MiniBEST Universal RNA Extraction Kit (TaKaRa, Japan) according to the manufacture's protocol. Total RNA was quantified by a NanoDrop ND-2000 spectrophotometer (NanoDrop Technologies, Delaware, USA) at 260/230 and 260/280 nm. Then 400 ng RNA was reversely transcribed into 10 μL cDNA by using the PrimeScriptTM RT Master Mix (TaKaRa, Japan) and the Peltier Thermal Cycler 200 (MJ Research, Watertown, MA, USA). The cDNA was dissolved in RNase-free water and stored at −20°C.

The two-step qRT-PCR reactions were performed in triplicate on 96-well plates using a 7500 Real-time PCR system (Applied Biosysytems, Foster, CA) with the SYBR® Premix Ex TaqTM (TaKaRa, Ostu, Japan). LBP (Lukkari et al., 1999), CD14 (Järveläinen et al., 1997) and β-actin primer sequences were synthesized by Sangon Biotech (Shanghai, China). These primer sequences were listed in Table 1. The concentrations of template and primers, the efficiency and consistency of LBP, CD14, and β-actin amplification were evaluated by a relative standard curve by echelon dilution (1:1–1:625). The reaction solution (20 μL) contains 10 μL SYBR® Premix Ex Taq, 0.4 μL PCR forward primer (10 μM) and 0.4 μL PCR reserve primer (10 μM), 0.4 ROX reference dye II, 2 μL cDNA and 6.8 μL dH_2_O. Cycling conditions were as follows: 30 s for denaturation at 95°C, 40 cycles of 5 s at 95°C and 34 s at 60°C for denaturation, followed by triple alternations between 95 and 60°C for melting curve analysis to verify the specificity of a single amplification. Fold changes of LBP and CD14 expression were calculated by the 2^−ΔΔCt^ method normalized to β-actin, setting soy protein group as the control.

**Table 1:** Primers used for qRT-PCR

**Gene**	**Primer**	**Sequence (5′–3′)**
LBP	Forward	GAGGCCTGAGTCTCTCCATCT
	Reverse	TCTGAGATGGCAAAGTAGACC
CD14	Forward	TGGAGCACGTACCTAAAGGG
	Reverse	GAGCTGTGGCTATGACTACGC
β-actin	Forward	ACCACAGCTGAGAGGGAAATCG
	Reverse	AGAGGTCTTTACGGATGTCAACG

### Western blotting

Liver protein was extracted using a commercial protein extraction kit (Thermo Pierce, NO. 78510). Whole protein was quantified with an Enhanced BCA Protein Assay Kit (Biyuntian, China). The liver proteins (60 μg per lane) were probed with anti-LBP antibody (Abcam, No. ab25094), anti-CD14 antibody (Abcam, No. ab182032) and β-Actin antibody (Santa Cruz, No. SC-47778). Three volumes of protein solution were combined with one volume loading buffer. Sixty micrograms of proteins were loaded onto 10% SDS–PAGE gels. Electrophoresis was performed at 60 V for 2 h at 4°C. Then the proteins were blotted by electrodiffusion for 2 h at 100 V on nitrocellulose membranes. The membranes were blocked with 5% skim milk in T-TBS for 1 h at room temperature. The membranes were then incubated overnight at 4°C with primary antibody in T-TBS containing 5% skimmed milk powder. Then the membranes were rinsed in T-TBS for 5 min and repeated for four times. After that, the membranes were incubated for 1 h with goat anti-rabbit IgG (H+L) (Thermo Pierce, No. 31160) or goat anti-mouse IgG (H+L) (Thermo Pierce, No. 31210) secondary antibodies, and rinsed in T-TBS for 5 min and repeated five times. Finally, blots were detected using SuperSignal® West Dura Extended Duration Substrate according to the manufacture's protocol. The western blot images were analyzed by using the Quantity One software (Biorad).

### Statistical analyses

Linear discriminant analysis (LDA) coupled with effect size measurements (LEfSe) was performed (http://huttenhower.sph.harvard.edu/galaxy/) to discover highly-dimensional gut bacteria and characterize the differences between two or more biological conditions (or classes; Segata et al., 2011; Zhu et al., 2015). The different features were identified at the OTU and genus levels.

Multivariate analyses were performed with the SIMCA-P software (version 11.5) to discriminate metabolites in colonic contents. Principal component analysis (PCA) and partial least squares discriminant analysis (PLS-DA) were performed on the NMR data. PLS-DA models were applied with five-fold cross-validation and evaluated with the R^2^X and Q^2^-values. The models were further validated with a permutation test (200 permutations). In orthogonal projection to latent structure (OPLS) model, X matrix represents for the concentration of all metabolites in each sample, and Y matrix represents for the group information of each sample. It can filter out the noise in data and distinguish the difference between two groups (Trygg and Wold, 2003), so it was preformed to maximize the separation between two groups. The metabolites were differentiated on the basis of variable importance in projection (VIP) scores with more than 1 and statistically significant change (*t*-test, *P* < 0.05) was considered to be responsible for the difference between two groups (Calvani et al., 2014).

Differences in other measurements between any two groups were evaluated by one-way analysis of variance (one-way ANOVA), and means were compared by Duncan's multiple comparison under the SAS system (version 9.2), *p* < 0.05 was declared significant.

## Results

### The composition of colonic polar metabolites varied by diets

A total of 67 different compounds were identified from all colonic contents based on ^1^H NMR spectrometry (Supplementary Table 1), including 22 amino acids, 7 short chain fatty acids, 8 sugars, 4 phenolic acids, 4 amines, 2 alcohols, 2 amino acid derivatives, 2 ketones, 5 nucleic acid components, 9 other organic acids, 1 vitamin/cofactor and choline.

**Figure 1:**
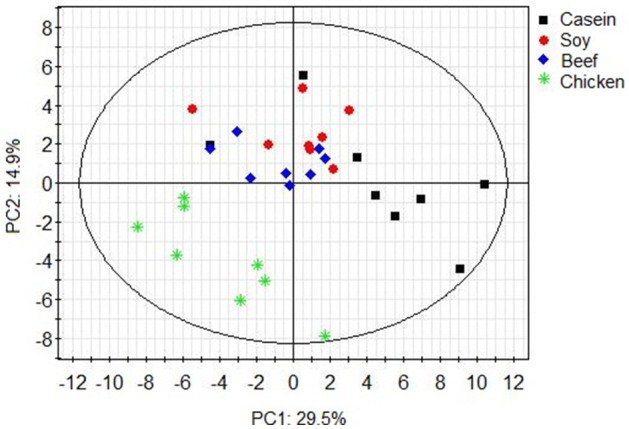
PCA scores plot of colonic metabolite profile of rats in response to different dietary proteins. Each point represents one biological sample.

Principle component analysis revealed great inter- and intra-group variations in metabolites (Figure 1). The chicken protein group was well-separated from the casein, beef, and soy protein groups, indicating that colonic metabolites showed different responses to chicken protein in the diet (*P* < 0.05) from those to casein, beef protein, or soy protein. The soy and beef protein groups showed a great similarity.

**Figure 2:**
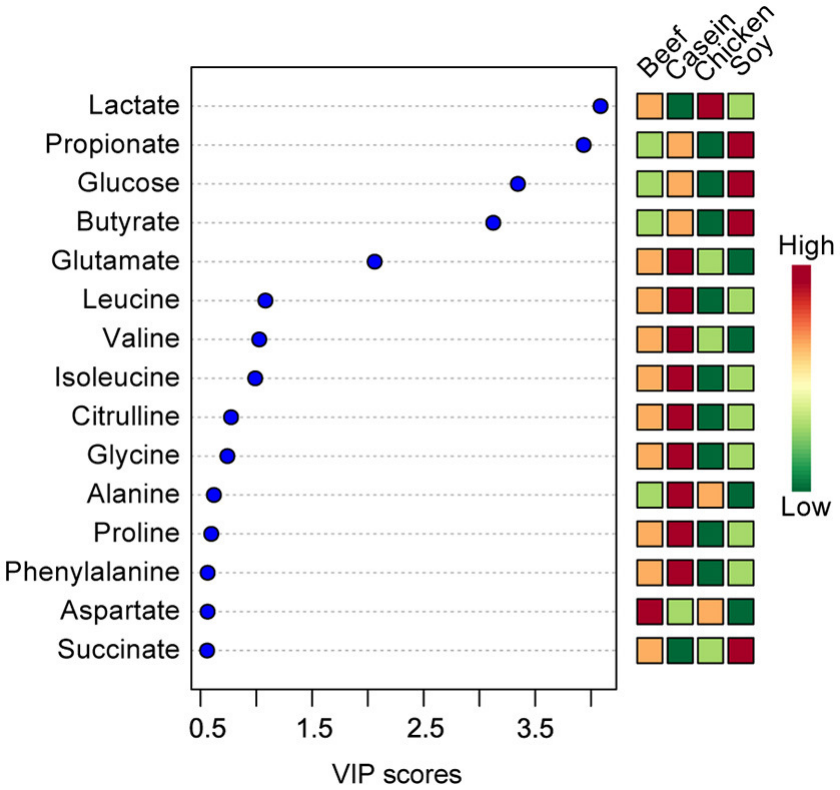
The top 15 VIP scores of component 1. The left part lists significant difference of metabolites; The middle part shows the top 15 VIP scores; The right heatmap shows the concentration of metabolites.

The top 15 VIP scores of component 1 were listed in Figure 2. The results showed that the rats fed with soy protein had the highest concentrations of propionate, glucose and butyrate. We also found that the soy protein group had higher levels of short chain fatty acids (923, 779, 666, and 645 μmol/L for the soy, casein, beef, and chicken protein groups, respectively, *P* < 0.05). The chicken protein group had the highest lactate, but the lowest for the casein group (1,704 vs. 217 μmol/L, respectively, *P* < 0.05). On the other hand, the casein group had the highest levels of amino acids (leucine, valine, isoleucine), while the chicken protein group had the lowest levels of these amino acids (*P* < 0.05).

**Figure 3:**
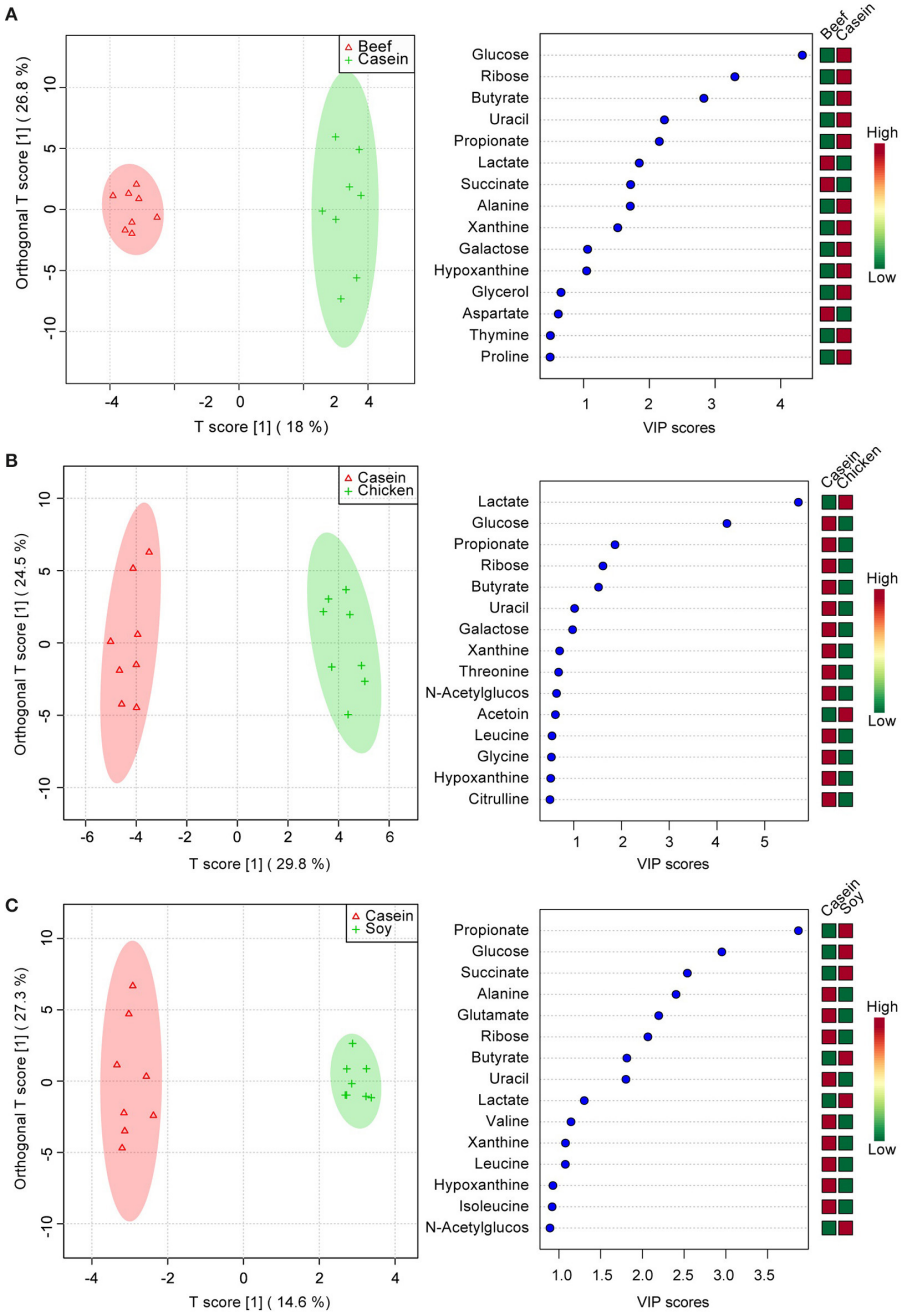
Pairwise comparisons between colonic contents extract spectra obtained from the beef, chicken and soy protein groups using OPLS analysis. Each figure has two parts: the left part is OPLS score plot, the right part is top 15 VIP scores. **(A)** beef protein group vs. casein group; **(B)** soy protein group vs. casein group; **(C)** chicken protein group vs. casein group.

To identify the effect of dietary proteins on colonic polar metabolites, pairwise comparison analysis was performed using OPLS between the casein group and one of the other three groups. One PLS component and one orthogonal component were calculated for all of the models. ^1^H NMR spectral data were used as the X matrix and classification information was used as the dummy Y matrix. The OPLS plot showed that the overall profile of colonic polar metabolites differed significantly (Figure 3). The responsible variables with top 15 VIP scores between the casein and the other three protein groups were showed in Figure 3. Compared to the casein group, the beef protein group had lower levels of glucose, ribose, galactose, butyrate, propionate, uracil, alanine, but higher concentrations of succinate and lactate. The chicken protein group had higher lactate but lower galactose, uracil, butyrate, ribose, propionate, and glucose. The soy protein group had higher succinate, glucose, propionate, lactate, and butyrate, but lower leucine, xanthine, valine, uracil, ribose, glutamate, and alanine.

### Gut microbiota had a distinct response to dietary proteins

#### General information

The 32 colonic samples had a total of 998,150 usable raw reads with an average of 31,192 ± 4,955 reads each (Supplementary Figure 1A). The reads were delineated into 837 operation taxonomy units (OTUs) with an average of 380 ± 70 per sample at a similarity level of 97% (Supplementary Figure 1B). No significant difference was observed in reads between any two diet groups (*p* > 0.05), but the beef protein group had a greater number of OTUs than the casein and chicken protein groups (*p* < 0.05). The rarefaction curves did not reach a stable state (Supplementary Figure 1C), but the Shannon–Wiener diversity estimates of all samples reached their plateaus (Supplementary Figure 1D), suggesting that the diversity of gut bacteria got stable. The Good's coverage index reached an average of 99.73 ± 0.06%, indicating the sequencing methodology was feasible. One biological sample in beef protein group was observed as an outlier as it had much smaller number of OTUs and lower Shannon–Wiener diversity estimate than the other samples. And thus the sample was excluded. There were no statistically significant differences (*P* > 0.05, Supplementary Table 2) among four groups in ACE, Chao, Shannon, Simpson, and Good's coverage indices for gut microbiota.

#### Diet effect

**Figure 4:**
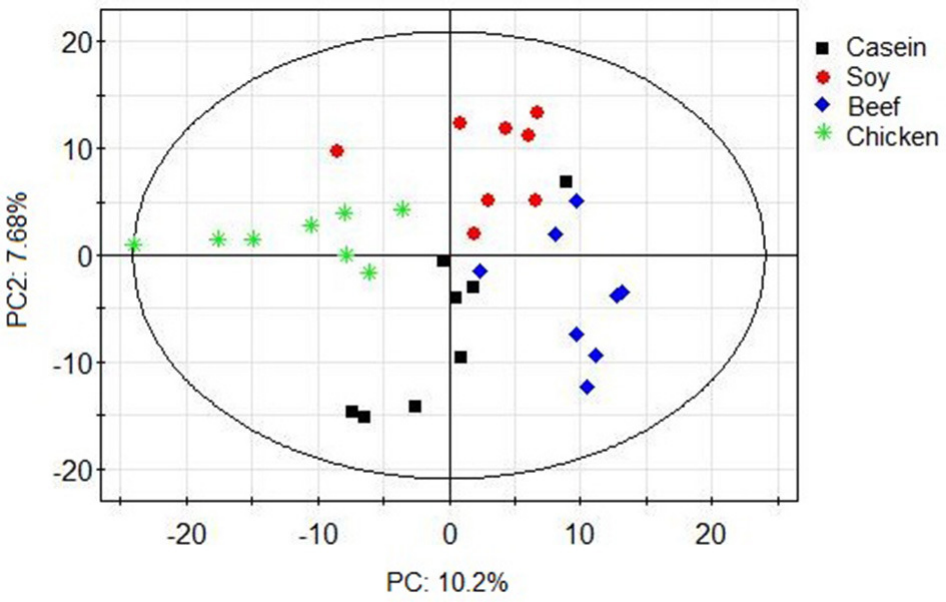
PCA scores plot of gut microbiota of rats in response to different dietary proteins. Each point represents one biological sample.

**Figure 5:**
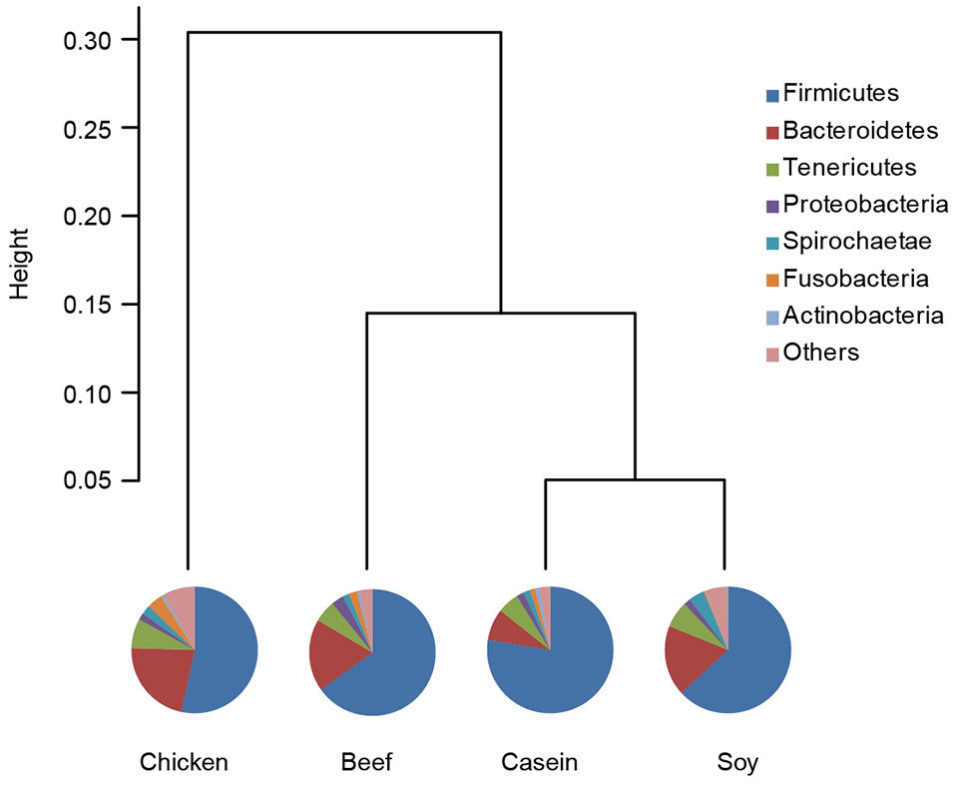
Relative abundance of gut microbiota at the phylum level. Pie chart showed shows the composition of gut microbiota at the phylum level. Clustering analysis shows the gut microbiota from the beef and soy protein group could be classified into the same subclass and separated from the chicken protein group.

Principle component analysis revealed great significant differences in colonic bacteria among diet groups (Figure 4). The chicken protein group was well-separated from the casein, beef, and soy protein groups in PC 1, while the chicken and beef protein groups were separated from the casein and soy protein groups in PC 2. The results indicate that gut bacteria showed different responses to chicken protein in the diet from those to casein, beef protein and soy protein. The soy and casein protein groups showed a great similarity. At phylum level (Figure 5), *Firmicutes* and *Bacteroidetes* were the two most predominant phyla for the four groups, contributing to 83.5, 75.5, 85.6, and 81.2% of variations the for casein, beef, chicken, and soy protein groups, respectively. Chicken protein group had the highest abundance of *Bacteroidetes*, but the lowest abundance of *Firmicutes*. Clustering analysis of gut bacteria at the phylum level showed that the gut microbiota from the beef, casein, and soy protein groups could be classified into the same subclass which was separated from those of the chicken protein group.

**Figure 6:**
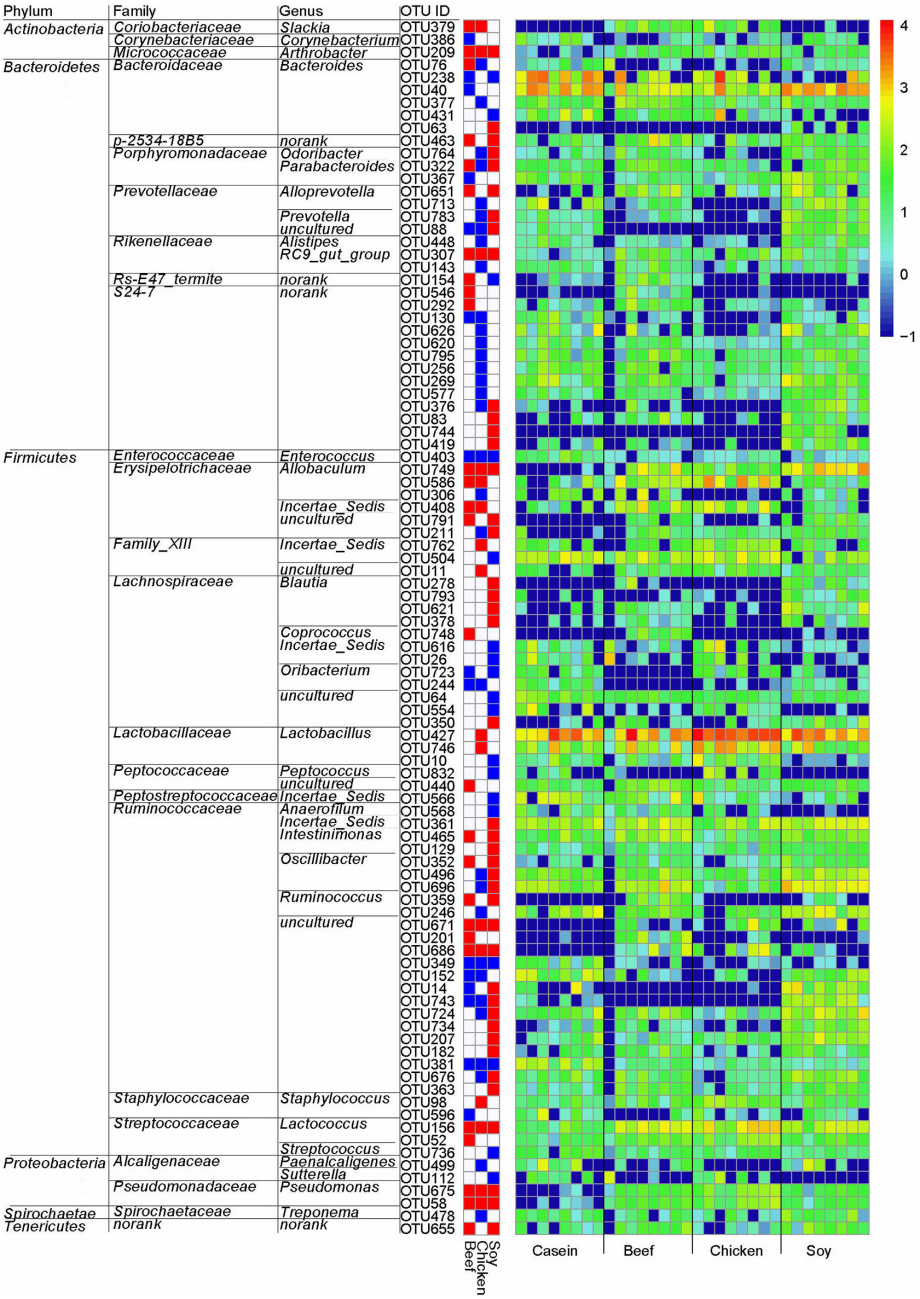
Gut bacteria at the OTU level in response to dietary proteins using LefSe. (1) The left part lists significant difference of OTU and corresponding phyla, families and genera; (2) The middle heatmap shows rich group and poor group of each OTU; (3) The right heatmap shows the relative abundance of OTU (log 10 transformed). Each column represents one biological sample and each row represents one OTU.

LeFSe analysis was performed on the OTU level to identify specific bacteria for different diet groups. Compared to the casein group, there were 96 differential OTUs (Figure 6). Of these OTUs, 16, 12 and 40 OTUs were higher in the beef, chicken and soy protein groups, respectively, and correspondingly, 15, 32, and 18 OTUs were lower in the above three groups, respectively. In particular, the chicken protein group had the highest relative abundance of OTUs for genus *Lactobacillus* (OTU427 and OTU746), while the soy protein group had the highest relative abundance of OTUs for family *Ruminococcaceae*.

### Dietary proteins affects the gut-derived endotoxins level in liver

LPS, gut-derived endotoxins, can bind to LBP in liver and activate Kupffer cells via CD14 receptor. Pro-inflammatory cytokines are released and this is postulated to promote liver injury.

**Figure 7:**
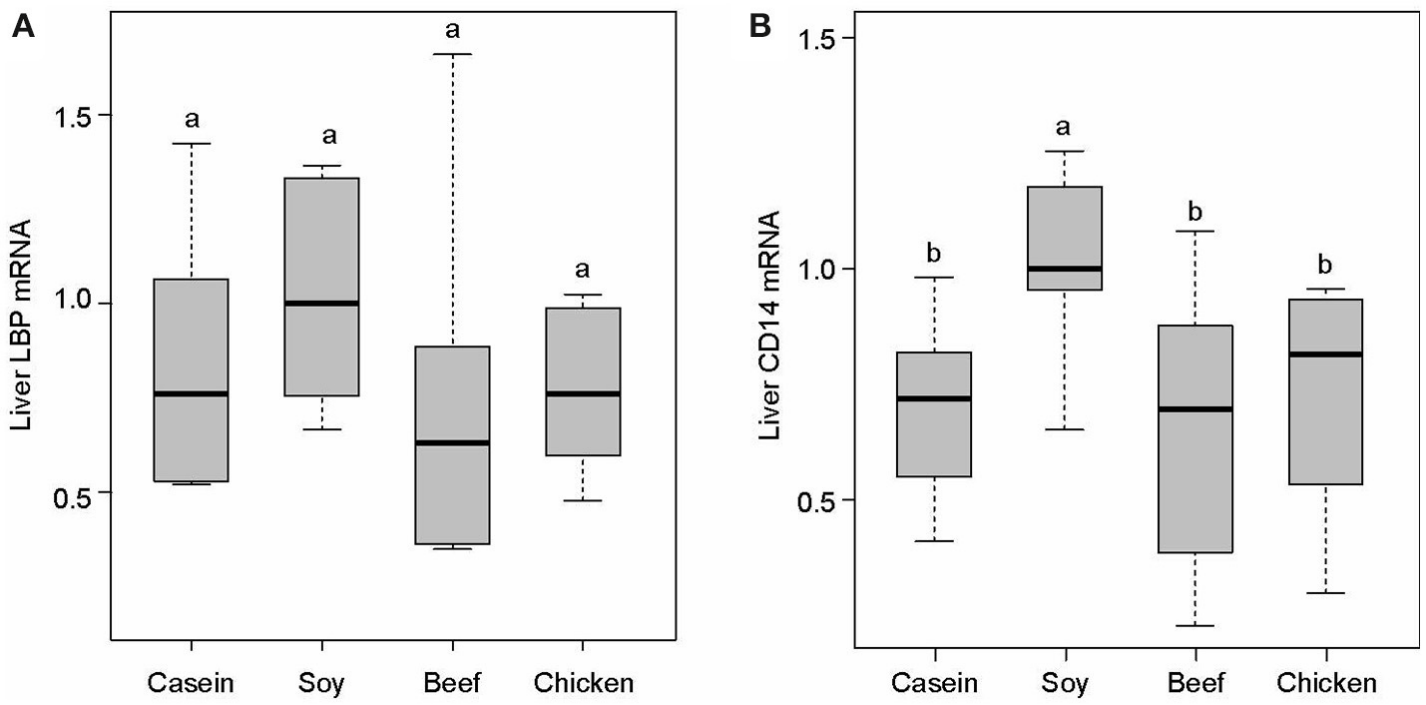
Gene expression levels of LBP **(A)** and CD14 **(B)** in the liver. All mRNA quantification data were normalized to the housekeeping gene β-actin. Gene expression levels were expressed as values relative to the soy protein group. Means with different superscripts differed significantly (*P* < 0.05).

**Figure 8:**
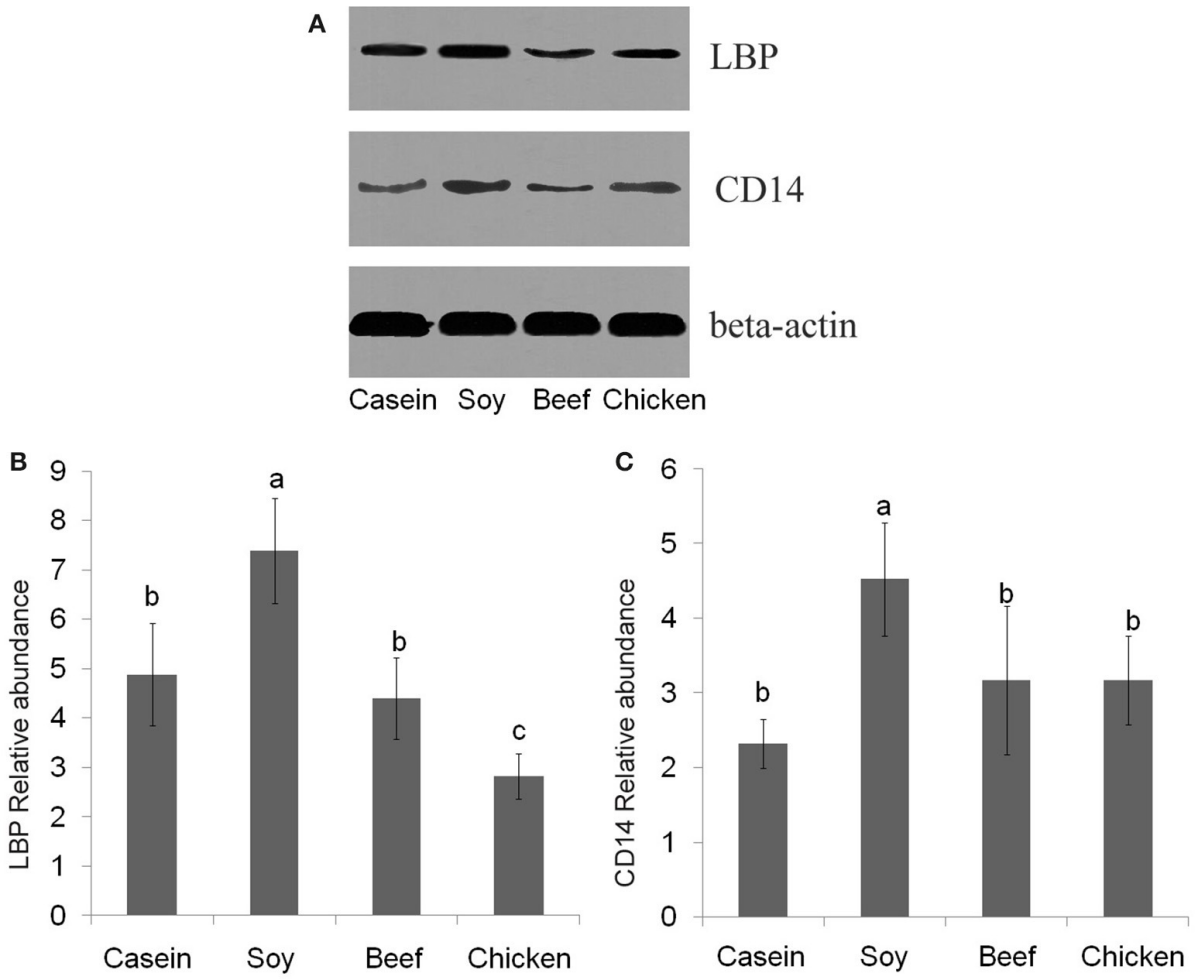
Western blotting profiles of LBP and CD14. **(A)** western blotting results; **(B)** LBP relative abundance; **(C)** CD14 relative abundance. a,b,c, Means with different letter differed significantly (*P* < 0.05).

No significant difference was found in LBP mRNA level among dietary groups (*P* > 0.05, Figure 7A). However, the levels of CD14 mRNA were found to be significantly lower in the casein, beef, and chicken protein groups than the soy protein group (*P* < 0.001, Figure 7B). The profile of LBP and CD14 proteins expression were detected by Western blotting. The results indicated that the LBP and CD14 protein levels were significantly higher in the soy protein group than any other protein group (*P* < 0.05, Figures 8A–C). The expression of LBP protein level was significantly lower in chicken protein group than in casein and beef protein groups (*P* < 0.05, Figures 8A,B), while no significant difference in CD14 protein level was observed between any two of casein, beef, and chicken protein groups (*P* > 0.05, Figure 8).

## Discussion

Gut microbiota plays a crucial role in human nutrition and health. Food components are usually digested in the stomach and small intestine, but the indigestible food compounds and endogenous proteins would enter into the large intestine for microbial fermentation and putrefaction, which shape diverse gut microbiota and metabolites (Van Hylckama Vlieg et al., 2011; Ridaura et al., 2013; Rist et al., 2013). The present study showed a significant impact of dietary proteins on gut microbiota and metabolites in rats.

*Firmicutes* and *Bacteroidetes* were observed to be the prevalent phyla in all samples. The casein, beef and soy protein groups had lower ratios of *Firmicutes* to *Bacteroidetes* (F/B ratio) than the chicken protein group (Supplementary Figure 3). The F/B ratio in the gut has been shown to be associated with obesity for both human and model animals (Turnbaugh et al., 2006, 2008, 2009; Turnbaugh and Gordon, 2009). However, all animals in the present study did not show obese characteristics. An interesting observation was that the body weight of one rat in beef protein group showed significant decrease during the last 2 weeks of feeding (Lin et al., 2016). The F/B ratio was the lowest (0.11 vs. 4.8% for the average of other seven rats in beef protein group; Supplementary Data 1).

Compared to the casein group, rats fed with chicken protein had higher level of beneficial genus *Lactobacillus*, while the soy protein group had the lowest abundance of this bacterium. *Lactobacillus* has been considered as a key player in host metabolic balance (Zhang et al., 2009; Arora et al., 2012). Higher abundance of *Lactobacillus* may reduce the antigen load from gut bacteria to the host, and alleviate inflammation responses and metabolic syndromes (Cani et al., 2007; Zhao, 2013). LBP could be as a biomarker of host inflammatory response and antigen load in blood (Zweigner et al., 2006). Our previous data showed that intake of meat proteins reduced serum LBP level compared to intake of soy protein (Zhu et al., 2015). In the present study, the chicken protein group had the highest concentration of lactate, which is in accordance with the changes of *Lactobacillus* abundance. The existence of *Lactobacillus* helps to maintain a high level of lactic acid, which would promote the lactate-utilizing species to thrive (Ruth et al., 2006; Chaucheyras-Durand and Duran, 2009). Western blotting results showed that LBP and CD14 were up-regulated in the rats fed with soy protein, as compared to the casein diet group. The chicken and beef protein diet did not have such an effect. In addition, the protein levels of glutathione S-transferases (*GSTs*), which involve detoxification or defense responses (Kim et al., 2004), were two-fold higher in the soy protein group than the other two groups (Supplementary Figure 2), which was in accordance with the results of LBP mRNA and CD14 mRNA in the liver (Zhu et al., 2015). LBP is an acute phase protein, and pro-inflammatory cytokines can increase LBP level (Lukkari et al., 1999). Our results suggested that intake of chicken protein at a normal dose may be more beneficial for the proliferation of commensal bacteria, as compared to the soy protein group.

Amino acid concentrations in colonic contents were much higher for the casein and beef protein groups than those for the chicken and soy protein groups. This could be attributed to several aspects. Firstly, some gut bacteria have the capacity of utilizing undigested dietary proteins and producing amino acids by excreting proteolytic enzymes. These bacteria belong to *Clostridium, Fusobacterium*, and *Acidaminococcus*. As shown above, genus *Fusobacterium* was higher in the casein and beef protein groups than the other two groups. Secondly, the composition of dietary proteins could also have a certain influence on microbial fermentation and the levels of amino acids in gut (Nocek et al., 2002; Nocek and Kautz, 2006). We also monitored the levels of amino acids in blood, and found that the rank of the levels of all amino acids was chicken protein group > soy protein group > beef protein group (Xuebin Shi, personal communication). This result showed that chicken protein may be easier to digest and absorb in the small intestine than soy and beef proteins, which caused few amino acids to enter into the large intestine (Christensen, 1984). Thirdly, the absorption activity of gut epithelium might have an impact on the levels of amino acids (Zhao et al., 2011). Therefore, beef and soy proteins could be less digested and absorbed in the small intestine and modify the composition of gut bacteria in the large intestine, which results in higher levels of amino acids in colonic contents. The underlying mechanism needs further investigations.

The intake of meat proteins and casein could reduce the fermentation of non-digestive fibers in rat colon. This was reflected by lower levels of short-chain fatty acids, glucose, N-acetylglucosamine, galactose, and ribose in colonic contents for the casein, beef, and chicken protein groups. Cornstarch and dextrinized cornstarch in diets were rich in dietary fibers and glycans which could not be degraded in both stomach and small intestine because of lacking specific enzymes in the host (Gill et al., 2006). However, there were at least 81 different glycoside hydrolase families in gut bacteria that contain genes involved in starch and sucrose metabolism, and the metabolism of glucose, galactose, fructose, arabinose, mannose, and xylose (Nocek et al., 2002). Previous studies showed that resistant starch diets could increase the abundance of *Ruminococcaceae* phylotypes in the gut (Walker et al., 2010; Salonen et al., 2014). Our results also showed that soy protein group had higher relative abundance of *Ruminococcaceae* (OTU 584, OTU682, OTU724, OTU779) that had a positive correlation with the levels of glucose, galactose, and ribose. This indicates that the intake of soy protein may favor the colonization of gut bacteria that have the capacity of degrading glycans. In addition, gut bacteria can utilize undigested carbohydrates, proteins, peptides, and amino acids to produce short-chain fatty acids that are energy source (especially butyrate) for colonocytes (Nicholson et al., 2012). Short-chain fatty acids have been shown to be associated with health benefits including glucose homeostasis, lipid metabolism, and reduced colon cancer risk (Byrne et al., 2015). The levels of butyrate and monosaccharide were associated positively with the relative abundances of *Alistipes, Prevotella, Alloprevotella*, and *Oscillibacter* (Zhao et al., 2013). *Prevotella* has the capability of utilizing a wide range of substrates and is a critical propionate producer (Reichardt et al., 2014). Although, the VIP score of propionate was <1, the level of propionate was higher in the soy protein group than those of the casein, beef, and chicken protein groups (141, 106, 85, and 58 μmol/L for the soy, casein, beef, and chicken protein groups, respectively). Gut microbiota can help the host get more energy from foods by fermenting undigested food ingredients and endogenous proteins to produce SCFAs (Cummings et al., 1987; Wong et al., 2006). This may explain the phenomenon that the soy protein group had lower body weight and weight gain, but higher visceral fat content (Zhu et al., 2015).

Based on the above results, soy protein intake could induce more carbohydrate metabolism as compared to beef and chicken proteins. Most of the amino acids (AAs) and their related metabolites were more abundant in casein and beef protein groups. The aromatic amino acids, such as phenylalanine and branched-chain amino acids, including valine, leucine, and isoleucine, were also higher in casein protein group. This could be attributed to higher abundance of gut bacteria such as *Fusobacterium*, which has the capacity of utilizing undigested dietary proteins and producing amino acids by excreting proteolytic enzymes.

## Conclusion

The type of proteins in diets had a significant impact on the compositions of gut bacteria and metabolites. Chicken protein promoted the growth of genus *Lactobacillus*, while soy protein promoted the growth of family *Ruminococcaceae*. Compared to meat proteins, the intake of soy protein may increase the degradation of dietary fibers and glycans and produce higher levels of short chain fatty acids. The casein and beef protein groups had higher levels of amino acids than the chicken protein group. Although the soy protein group had higher levels of SCFAs, the relative abundance of beneficial bacteria was lower, and the detoxification or defense responses related proteins in host liver were higher than meat protein groups. Meanwhile, long-term intake of soy protein led to the up-regulation of CD14 and LBP in liver and the level of LBP in serum (The result about LBP in serum was shown in reference 24, our previously published paper), suggesting that bacterial endotoxins were elevated. Our results confirmed that long-term intake of meat proteins can maintain a more balanced composition of gut bacteria and reduce the antigen load and inflammatory response from gut bacteria to the host.

## Additional information

Sequence information: all sequence data have been deposited in the NCBI Sequence Read Archive under accession code SRP066996.

## Author contributions

The seven authors are justifiably credited with authorship, according to the authorship criteria. YZ: Design, acquisition of data, analysis, and interpretation of data, drafting the manuscript, final approval given; CL: Conception, design, partial acquisition of data, analysis, and interpretation of data, drafting the manuscript, funding holder, final approval given; XL: Data acquisition of animal rearing, final approval given; XX: Critical revision of manuscript, final approval given; XS: Data analysis, final approval given; KY: Acquisition of data, final approval given; GZ: Conception, design, analysis and interpretation of data, critical revision of the manuscript, final approval given.

### Conflict of interest statement

The authors declare that the research was conducted in the absence of any commercial or financial relationships that could be construed as a potential conflict of interest.

## Supplementary material

Supplementary Figure 1 Diversity estimation of ceacel microbiota in all samples. **(A)** The average number of usable raw reads (mean and standard deviation). One-way ANOVA and Duncan's multiple comparisons indicated that pork protein groups had higher abundance of usable raw reads than soy and chicken protein groups (*p* < 0.05); **(B)** The average number of OTU (mean and standard deviation). One-way ANOVA indicated that there was no significant difference between any two diet groups in OTU number (*p* > 0.05); **(C)** Rarefaction curves. Each line represents one rat; **(D)** Shannon–Wiener diversity index curves. Each line represents one rat.

Supplementary Figure 2 The expression of glutathione S-transferases in liver.

Supplementary Figure 3 The F/B ratio of rats fed different kinds of dietary proteins. a,b, Means with different letter differed significantly (*P* < 0.05).

Supplementary Table 1 The composition of metabolites profile.

Supplementary Table 2 Richness and diversity indexes relative to each sample.
