# Supplementary material for: Beef, Chicken, and Soy Proteins in Diets Induce Different Gut Microbiota and Metabolites in Rats
Source: Front Microbiol. 2017 Jul 27;8:1395. doi: 10.3389/fmicb.2017.01395 (PMC5530634; doi:10.3389/fmicb.2017.01395)
Supplement: Supplementary Figure 1 — Diversity estimation of ceacel microbiota in all samples. (A) The average number of usable raw reads (mean and standard deviation). One-way ANOVA and Duncan's multiple comparisons indicated that pork protein groups had higher abundance of usable raw reads than soy and chicken protein groups (p < 0.05); (B) The average number of OTU (mean and standard deviation). One-way ANOVA indicated that there was no significant difference between any two diet groups in OTU number (p > 0.05); (C) Rarefaction curves. Each line represents one rat; (D) Shannon–Wiener diversity index curves. Each line represents one rat. [file Image1.PDF]

# Supplementary Figure

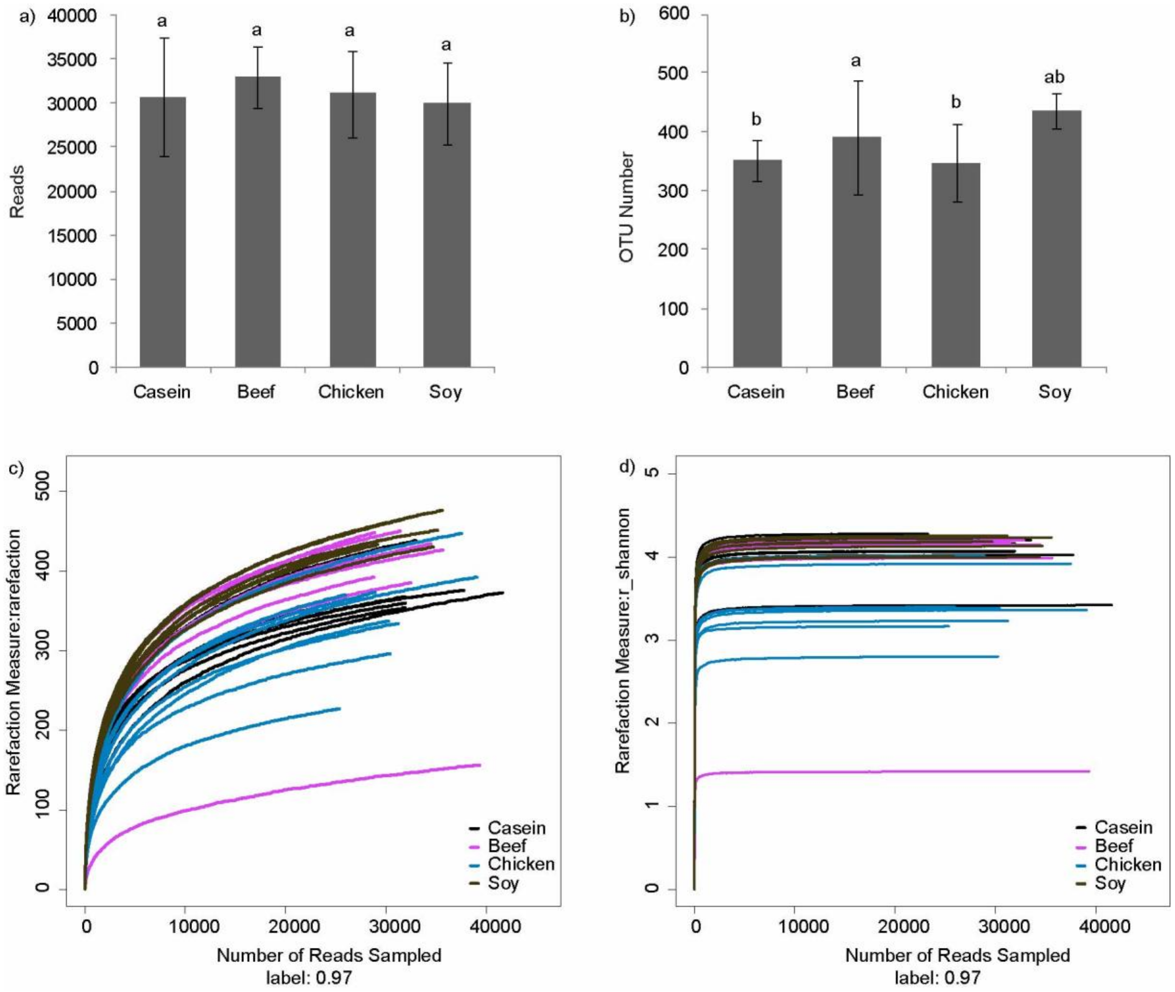


**Supplementary Figure S1 Diversity estimation of ceacel microbiota in all samples.**

a) The average number of usable raw reads (mean and standard deviation). One-way ANOVA and Duncan's multiple comparisons indicated that pork protein groups had higher abundance of usable raw reads than soy and chicken protein groups ($p<0.05$);

b) The average number of OTU (mean and standard deviation). One-way ANOVA indicated that there was no significant difference between any two diet groups in OTU number ($p>0.05$);

c) Rarefaction curves. Each line represents one rat;

d) Shannon–Wiener diversity index curves. Each line represents one rat;
