# Supplementary figures and images for: Beef, Chicken, and Soy Proteins in Diets Induce Different Gut Microbiota and Metabolites in Rats
Source: Front Microbiol. 2017 Jul 27;8:1395. doi: 10.3389/fmicb.2017.01395 (PMC5530634; doi:10.3389/fmicb.2017.01395)

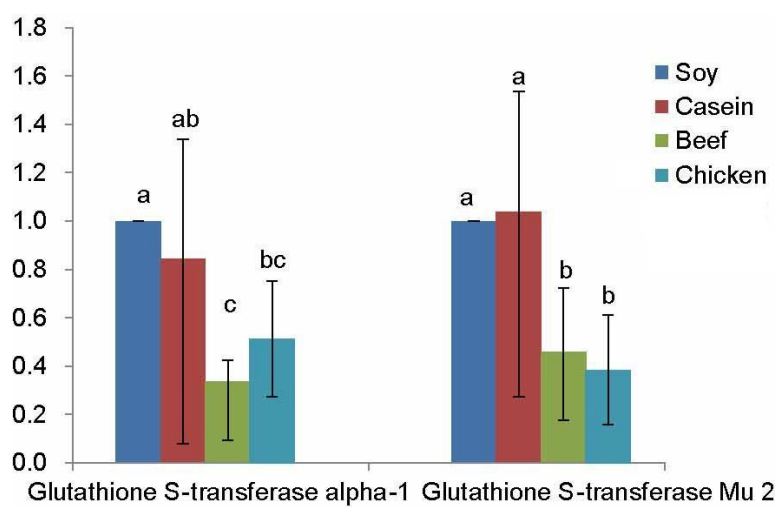

**Supplementary Figure S2 The expression of glutathione S-transferases in liver**

Supplement: Supplementary Figure 2 — The expression of glutathione S-transferases in liver. [file Image2.PDF]

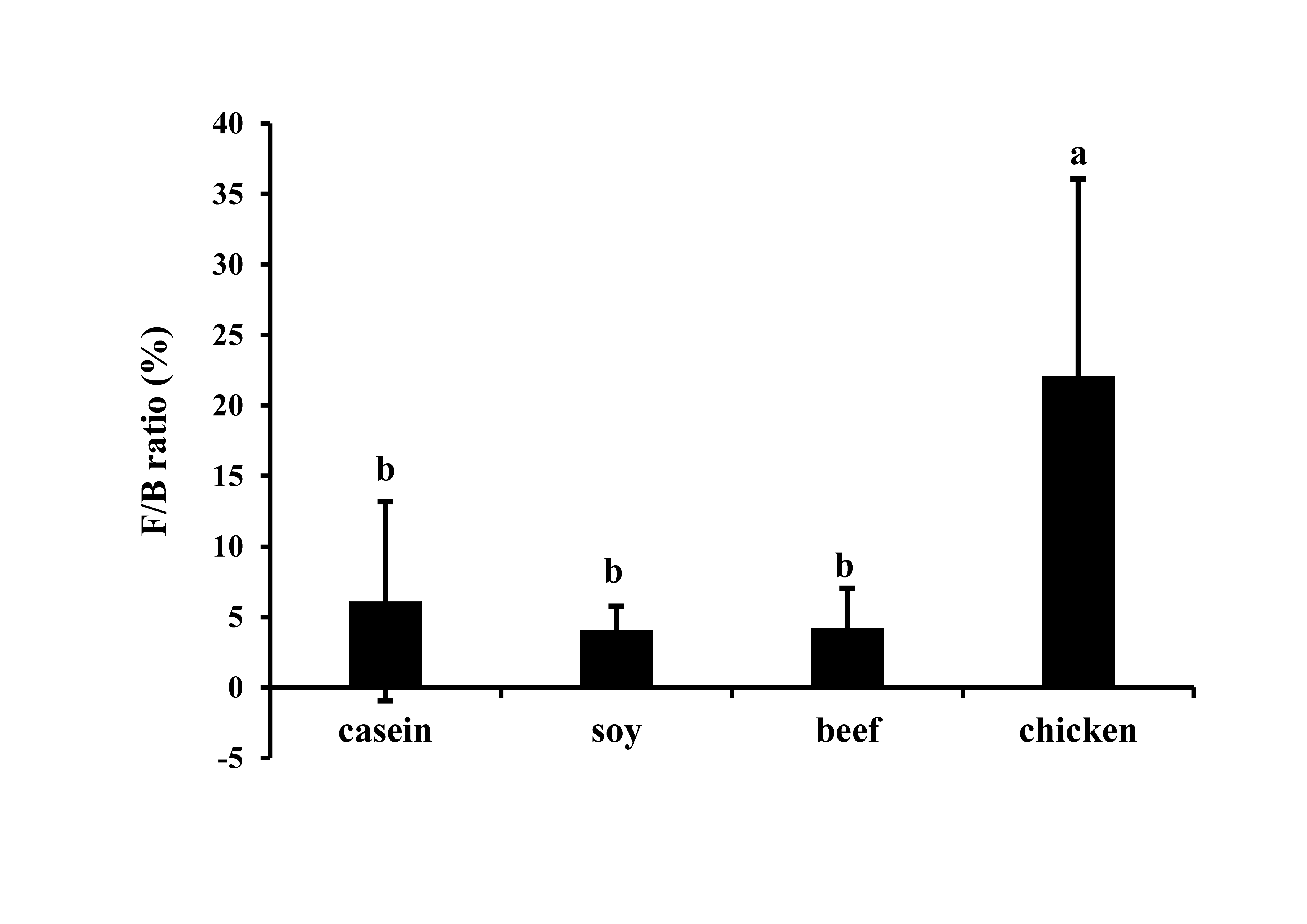

Supplement: Supplementary Figure 3 — The F/B ratio of rats fed different kinds of dietary proteins. a,b, Means with different letter differed significantly (P < 0.05). [file Image3.TIF]
