# Supplementary material for: Beef, Chicken, and Soy Proteins in Diets Induce Different Gut Microbiota and Metabolites in Rats
Source: Front Microbiol. 2017 Jul 27;8:1395. doi: 10.3389/fmicb.2017.01395 (PMC5530634; doi:10.3389/fmicb.2017.01395)
Supplement: Supplementary Table 1 — The composition of metabolites profile. [file Table1.DOC]

**Supplementary Table S1 The composition of metabolites profile**

| Metabolite  types | Metabolite names | Concentration(μmol/L, mean ±standard deviation) | | | | | | | | | | | |
| --- | --- | --- | --- | --- | --- | --- | --- | --- | --- | --- | --- | --- | --- |
|  |  | Casein | | | Beef | | | Chicken | | | Soy | | |
| Alcohols | Ethanol | 3563 | ± | 6043 | 1319 | ± | 1704 | 1679 | ± | 1509 | 1970 | ± | 3081 |
|  | Methanol | 198 | ± | 17 | 186 | ± | 11 | 175 | ± | 14 | 216 | ± | 17 |
| Amines | Creatinine | 13 | ± | 5 | 9 | ± | 4 | 10 | ± | 4 | 11 | ± | 3 |
|  | Dimethylamine | 5 | ± | 3 | 4 | ± | 1 | 4 | ± | 1 | 4 | ± | 1 |
|  | Methylamine | 17 | ± | 9 | 39 | ± | 33 | 32 | ± | 8 | 21 | ± | 12 |
|  | Trimethylamine | 4 | ± | 3 | 3 | ± | 2 | 4 | ± | 3 | 4 | ± | 3 |
| Amino acid derivatives | Creatine | 25 | ± | 17 | 14 | ± | 6 | 18 | ± | 18 | 19 | ± | 11 |
|  | Pyroglutamate | 35 | ± | 25 | 21 | ± | 15 | 14 | ± | 11 | 21 | ± | 11 |
| Amino acids | 2-Aminobutyrate | 165 | ± | 58 | 146 | ± | 44 | 179 | ± | 65 | 143 | ± | 35 |
|  | Alanine | 954 | ± | 253 | 891 | ± | 128 | 990 | ± | 234 | 853 | ± | 105 |
|  | Aspartate | 146 | ± | 35 | 202 | ± | 56 | 202 | ± | 66 | 123 | ± | 37 |
|  | beta-Alanine | 8 | ± | 6 | 19 | ± | 25 | 13 | ± | 5 | 11 | ± | 4 |
|  | Betaine | 4 | ± | 2 | 5 | ± | 2 | 5 | ± | 2 | 4 | ± | 2 |
|  | Citrulline | 234 | ± | 97 | 225 | ± | 29 | 137 | ± | 43 | 189 | ± | 37 |
|  | Glutamate | 956 | ± | 227 | 1046 | ± | 133 | 976 | ± | 320 | 860 | ± | 161 |
|  | Glutamine | 81 | ± | 22 | 51 | ± | 26 | 32 | ± | 36 | 81 | ± | 18 |
|  | Glycine | 483 | ± | 141 | 481 | ± | 55 | 383 | ± | 94 | 451 | ± | 85 |
|  | Isoleucine | 308 | ± | 95 | 318 | ± | 55 | 212 | ± | 73 | 259 | ± | 48 |
|  | Leucine | 328 | ± | 106 | 338 | ± | 37 | 228 | ± | 60 | 274 | ± | 57 |
|  | Lysine | 328 | ± | 110 | 339 | ± | 83 | 363 | ± | 107 | 309 | ± | 50 |
|  | Methionine | 131 | ± | 44 | 136 | ± | 18 | 115 | ± | 34 | 113 | ± | 21 |
|  | Ornithine | 24 | ± | 12 | 23 | ± | 10 | 36 | ± | 19 | 23 | ± | 6 |
|  | Phenylalanine | 191 | ± | 63 | 191 | ± | 38 | 139 | ± | 29 | 158 | ± | 31 |
|  | Proline | 322 | ± | 93 | 305 | ± | 42 | 252 | ± | 88 | 278 | ± | 53 |
|  | Sarcosine | 17 | ± | 8 | 16 | ± | 5 | 9 | ± | 3 | 16 | ± | 2 |
|  | Serine | 199 | ± | 73 | 186 | ± | 59 | 126 | ± | 35 | 217 | ± | 28 |
|  | Threonine | 253 | ± | 91 | 241 | ± | 73 | 105 | ± | 65 | 249 | ± | 37 |
|  | Tryptophan | 16 | ± | 7 | 25 | ± | 9 | 19 | ± | 6 | 10 | ± | 7 |
|  | Tyrosine | 207 | ± | 67 | 196 | ± | 37 | 161 | ± | 37 | 177 | ± | 29 |
|  | Valine | 449 | ± | 126 | 470 | ± | 59 | 404 | ± | 121 | 395 | ± | 61 |
| Ammoniums compounds | Choline | 15 | ± | 12 | 10 | ± | 5 | 19 | ± | 23 | 12 | ± | 8 |
| Ketones | Acetoin | 30 | ± | 39 | 56 | ± | 56 | 190 | ± | 128 | 33 | ± | 39 |
|  | Acetone | 21 | ± | 13 | 8 | ± | 3 | 10 | ± | 7 | 9 | ± | 4 |
| Nucleic acid components | Hypoxanthine | 186 | ± | 82 | 109 | ± | 44 | 82 | ± | 23 | 134 | ± | 34 |
|  | Oxypurinol | 33 | ± | 54 | 36 | ± | 29 | 22 | ± | 32 | 5 | ± | 14 |
|  | Thymine | 70 | ± | 34 | 31 | ± | 18 | 41 | ± | 16 | 59 | ± | 13 |
|  | Uracil | 329 | ± | 143 | 165 | ± | 72 | 130 | ± | 40 | 234 | ± | 34 |
|  | Xanthine | 177 | ± | 93 | 61 | ± | 31 | 34 | ± | 27 | 121 | ± | 41 |
| Organic acids | 2-Oxobutyrate | 24 | ± | 13 | 17 | ± | 3 | 43 | ± | 11 | 22 | ± | 5 |
|  | 2-Oxocaproate | 25 | ± | 5 | 24 | ± | 8 | 21 | ± | 5 | 20 | ± | 8 |
|  | 2-Oxoglutarate | 86 | ± | 45 | 68 | ± | 22 | 122 | ± | 30 | 92 | ± | 22 |
|  | 2-Oxoisocaproate | 109 | ± | 50 | 80 | ± | 20 | 117 | ± | 26 | 108 | ± | 14 |
|  | Fumarate | 40 | ± | 9 | 39 | ± | 12 | 27 | ± | 11 | 42 | ± | 4 |
|  | Lactate | 257 | ± | 327 | 408 | ± | 363 | 1704 | ± | 956 | 333 | ± | 337 |
|  | Malonate | 26 | ± | 6 | 30 | ± | 21 | 18 | ± | 8 | 23 | ± | 5 |
|  | Pyruvate | 76 | ± | 30 | 71 | ± | 11 | 112 | ± | 27 | 66 | ± | 16 |
|  | Succinate | 53 | ± | 34 | 236 | ± | 442 | 83 | ± | 78 | 286 | ± | 711 |
| Phenolic acid | 3-Methyl-2-oxovalerate | 113 | ± | 55 | 87 | ± | 21 | 124 | ± | 30 | 98 | ± | 22 |
|  | 3-Phenylpropionate | 72 | ± | 29 | 55 | ± | 26 | 62 | ± | 15 | 56 | ± | 38 |
|  | 4-Hydroxyphenylacetate | 18 | ± | 14 | 10 | ± | 3 | 8 | ± | 5 | 21 | ± | 28 |
|  | 4-Hydroxyphenyllactate | 10 | ± | 8 | 7 | ± | 3 | 8 | ± | 4 | 9 | ± | 2 |
| SCFA | Acetate | 6277 | ± | 2187 | 5131 | ± | 1450 | 5177 | ± | 1977 | 6592 | ± | 2506 |
|  | Butyrate | 753 | ± | 289 | 433 | ± | 201 | 329 | ± | 86 | 887 | ± | 492 |
|  | Formate | 34 | ± | 9 | 29 | ± | 8 | 103 | ± | 94 | 27 | ± | 6 |
|  | Isobutyrate | 20 | ± | 20 | 8 | ± | 3 | 68 | ± | 11 | 14 | ± | 10 |
|  | Isovalerate | 100 | ± | 28 | 58 | ± | 17 | 64 | ± | 12 | 95 | ± | 46 |
|  | Propionate | 1382 | ± | 878 | 846 | ± | 468 | 582 | ± | 192 | 1414 | ± | 1001 |
|  | Valerate | 225 | ± | 111 | 154 | ± | 58 | 125 | ± | 25 | 202 | ± | 156 |
| Sugars | 1,3-Dihydroxyacetone | 16 | ± | 9 | 9 | ± | 6 | 1 | ± | 2 | 19 | ± | 7 |
|  | Fucose | 175 | ± | 72 | 163 | ± | 44 | 177 | ± | 70 | 175 | ± | 21 |
|  | Galactose | 428 | ± | 174 | 362 | ± | 132 | 216 | ± | 144 | 444 | ± | 58 |
|  | Glucose | 1375 | ± | 713 | 933 | ± | 257 | 283 | ± | 292 | 1644 | ± | 307 |
|  | Glycerol | 147 | ± | 79 | 86 | ± | 52 | 150 | ± | 245 | 101 | ± | 30 |
|  | N-Acetylglucosamine | 336 | ± | 148 | 393 | ± | 158 | 206 | ± | 110 | 453 | ± | 111 |
| Vitamin/  cofactors | Ribose | 527 | ± | 218 | 283 | ± | 112 | 203 | ± | 94 | 439 | ± | 116 |
|  | Xylose | 553 | ± | 222 | 623 | ± | 238 | 540 | ± | 183 | 683 | ± | 189 |
|  | Nicotinate | 33 | ± | 9 | 27 | ± | 6 | 27 | ± | 9 | 35 | ± | 10 |
