# Supplementary material for: Beef, Chicken, and Soy Proteins in Diets Induce Different Gut Microbiota and Metabolites in Rats
Source: Front Microbiol. 2017 Jul 27;8:1395. doi: 10.3389/fmicb.2017.01395 (PMC5530634; doi:10.3389/fmicb.2017.01395)
Supplement: Supplementary Table 2 — Richness and diversity indexes relative to each sample. [file Table2.DOC]

**Supplementary Table S2 Richness and diversity indexes relative to each sample**

| **Sample ID** | **Reads** | **0.97** | | | | | |
| --- | --- | --- | --- | --- | --- | --- | --- |
|  |  | **OTU** | **ace** | **chao** | **coverage** | **shannon** | **simpson** |
| Casein1 | 31786 | 365 | 435 | 456 | 0.997735 | 4.06 | 0.0417 |
|  |  |  | (409,478) | (415,533) |  | (4.04,4.08) | (0.0408,0.0425) |
| Casein2 | 22243 | 349 | 409 | 414 | 0.996853 | 4.05 | 0.0359 |
|  |  |  | (387,446) | (385,468) |  | (4.03,4.07) | (0.0351,0.0368) |
| Casein3 | 37596 | 373 | 414 | 405 | 0.998537 | 4.01 | 0.0382 |
|  |  |  | (398,443) | (389,435) |  | (3.99,4.02) | (0.0374,0.0389) |
| Casein4 | 23843 | 266 | 326 | 326 | 0.997232 | 2.39 | 0.2148 |
|  |  |  | (303,363) | (298,377) |  | (2.36,2.42) | (0.211,0.2187) |
| Casein5 | 31791 | 357 | 418 | 431 | 0.997924 | 4.15 | 0.0341 |
|  |  |  | (394,457) | (396,496) |  | (4.13,4.17) | (0.0334,0.0348) |
| Casein6 | 41430 | 370 | 434 | 432 | 0.998141 | 3.41 | 0.0709 |
|  |  |  | (411,471) | (405,481) |  | (3.39,3.42) | (0.0699,0.0719) |
| Casein7 | 31791 | 351 | 408 | 411 | 0.997892 | 4.05 | 0.0335 |
|  |  |  | (386,443) | (383,462) |  | (4.03,4.07) | (0.0329,0.0341) |
| Casein8 | 25157 | 375 | 442 | 440 | 0.996979 | 4.05 | 0.0388 |
|  |  |  | (417,480) | (411,491) |  | (4.03,4.07) | (0.0379,0.0398) |
| Beef1 | 32859 | 435 | 502 | 504 | 0.997 | 4.2 | 0.034 |
|  |  |  | (478,538) | (475,554) |  | (4.18,4.22) | (0.0332,0.0347) |
| Beef2 | 32474 | 385 | 446 | 444 | 0.998 | 3.36 | 0.1423 |
|  |  |  | (424,482) | (418,491) |  | (3.33,3.38) | (0.1388,0.1457) |
| Beef3 | 31440 | 450 | 523 | 548 | 0.997 | 4.17 | 0.0449 |
|  |  |  | (497,562) | (507,620) |  | (4.15,4.19) | (0.0436,0.0461) |
| Beef4 | 39337 | 156 | 254 | 238 | 0.999 | 1.42 | 0.476 |
|  |  |  | (222,302) | (195,328) |  | (1.4,1.44) | (0.4703,0.4816) |
| Beef5 | 28779 | 392 | 458 | 485 | 0.997 | 4.18 | 0.0328 |
|  |  |  | (434,496) | (444,559) |  | (4.17,4.2) | (0.0321,0.0335) |
| Beef6 | 34479 | 434 | 504 | 521 | 0.997 | 4.15 | 0.0436 |
|  |  |  | (479,542) | (484,584) |  | (4.13,4.17) | (0.0424,0.0448) |
| Beef7 | 28869 | 448 | 534 | 554 | 0.997 | 4.02 | 0.0466 |
|  |  |  | (506,577) | (511,625) |  | (4,4.04) | (0.0455,0.0477) |
| Beef8 | 35673 | 426 | 479 | 505 | 0.998 | 3.99 | 0.0432 |
|  |  |  | (459,510) | (470,570) |  | (3.97,4.01) | (0.0422,0.0441) |
| Chicken1 | 30264 | 337 | 412 | 408 | 0.997 | 2.8 | 0.1883 |
|  |  |  | (386,454) | (378,460) |  | (2.78,2.83) | (0.1843,0.1922) |
| Chicken2 | 30418 | 296 | 354 | 373 | 0.998 | 3.39 | 0.092 |
|  |  |  | (331393 | (337,442) |  | (3.37,3.41) | (0.0898,0.0941) |
| Chicken3 | 25367 | 227 | 276 | 276 | 0.998 | 3.17 | 0.0895 |
|  |  |  | (256,313) | (251,326) |  | (3.15,3.19) | (0.0876,0.0914) |
| Chicken4 | 25969 | 370 | 444 | 442 | 0.997 | 3.4 | 0.1159 |
|  |  |  | (418,485) | (411,496) |  | (3.37,3.42) | (0.113,0.1189) |
| Chicken5 | 28913 | 373 | 452 | 463 | 0.997 | 4.02 | 0.0378 |
|  |  |  | (424,496) | (424,530) |  | (4,4.03) | (0.0371,0.0386) |
| Chicken6 | 31247 | 334 | 393 | 434 | 0.998 | 3.23 | 0.088 |
|  |  |  | (371,430) | (389,517) |  | (3.21,3.25) | (0.0864,0.0895) |
| Chicken7 | 37516 | 447 | 524 | 536 | 0.998 | 3.92 | 0.0559 |
|  |  |  | (498,564) | (499,598) |  | (3.9,3.94) | (0.0547,0.0572) |
| Chicken8 | 39075 | 392 | 462 | 467 | 0.998 | 3.36 | 0.0928 |
|  |  |  | (437,501) | (435,525) |  | (3.34,3.38) | (0.0911,0.0944) |
| Soy1 | 29195 | 433 | 502 | 502 | 0.997 | 4.16 | 0.0372 |
|  |  |  | (478,539) | (473,552) |  | (4.14,4.18) | (0.0363,0.0381) |
| Soy2 | 25857 | 385 | 484 | 478 | 0.996 | 4.01 | 0.0458 |
|  |  |  | (451,535) | (440,542) |  | (3.99,4.03) | (0.0444,0.0472) |
| Soy3 | 34701 | 430 | 501 | 517 | 0.998 | 4.13 | 0.033 |
|  |  |  | (476,540) | (480,581) |  | (4.11,4.15) | (0.0324,0.0336) |
| Soy4 | 35597 | 476 | 550 | 547 | 0.998 | 4.24 | 0.0315 |
|  |  |  | (525,589) | (518,598) |  | (4.22,4.25) | (0.0309,0.0321) |
| Soy5 | 34409 | 473 | 545 | 566 | 0.997 | 4 | 0.0474 |
|  |  |  | (520,582) | (528,630) |  | (3.98,4.02) | (0.0464,0.0484) |
| Soy6 | 27636 | 433 | 527 | 522 | 0.997 | 4.18 | 0.0348 |
|  |  |  | (495,574) | (486,583) |  | (4.16,4.2) | (0.034,0.0356) |
| Soy7 | 22746 | 412 | 497 | 507 | 0.996 | 4.13 | 0.0386 |
|  |  |  | (469,541) | (468,573) |  | (4.1,4.15) | (0.0376,0.0396) |
| Soy8 | 29693 | 439 | 528 | 517 | 0.997 | 4.19 | 0.0336 |
|  |  |  | (498,573) | (485,570) |  | (4.17,4.2) | (0.0328,0.0344) |
